# Two Cases of Squamous Cell Carcinoma of the Penis – A Dermoscopic View

**DOI:** 10.5826/dpc.1101a97

**Published:** 2020-12-10

**Authors:** Shekhar Neema, S. Radhakrishnan, Pratik Kinra, Sunmeet Sandhu

**Affiliations:** 1 Department of Dermatology, Armed Forces Medical College, Pune, India; 2 Department of Pathology, Armed Forces Medical College, Pune, India

**Keywords:** squamous cell carcinoma, penis, dermoscopy

## Introduction

Penile cancer is an uncommon cancer in western countries, accounting for 0.4%–0.6% of total malignancies. It is more common in Asian countries and consists of 6% of total malignancies in India. It is more common in uncircumcised men, and the prevalence increases with age. Phimosis, human papillomavirus, poor hygienic practice, uncircumcised state, lichen sclerosus et atrophicus (LSA), balanitis, penile trauma, and smoking are considered risk factors for penile cancer [1]. There is a delay in diagnosis, as it does not interfere with voiding and erectile function in its initial stage and the associated embarrassment due to its location.

## Case Presentation

**Figure 1:**
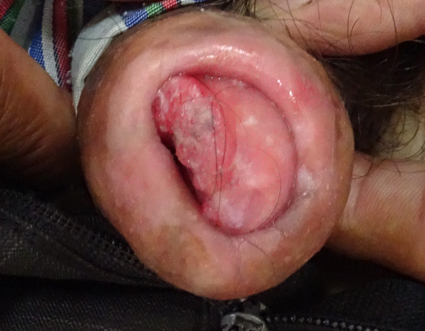
Ulceroproliferative growth measuring 2 × 2 cm over the right side of the glans penis. Phimosis and edema of the prepuce can be appreciated.

**Figure 2:**
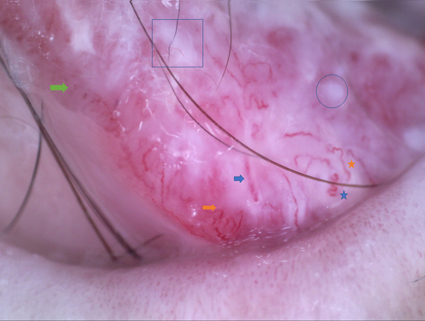
Dermoscopy shows dotted vessels (green arrow), linear vessels (blue arrow), hairpin vessels (orange arrow), corkscrew vessels (blue star), and serpentine vessels (orange star). White clods (blue circle) and white structureless areas (blue square) can be seen (Dino-Lite Edge, polarized ×100).

A 58-year-old male, who is a chronic tobacco chewer, presented with insidious onset of a gradually progressive painless ulceroproliferative growth over the glans penis of 1 year’s duration. There was history of difficulty with voiding and sexual intercourse over the previous 2 months. There was no history suggestive of LSA. Examination revealed phimosis and an ulceroproliferative growth measuring 2 × 2 cm over the glans penis. The growth was firm and displaced the urethral meatus (Figure 1). Bilateral inguinal lymphadenopathy was present. Dermoscopy of the lesion showed polymorphic vessels (linear, coiled, hairpin, dotted, and corkscrew), white clods, and structureless white areas (Figure 2.)

**Figure 3:**
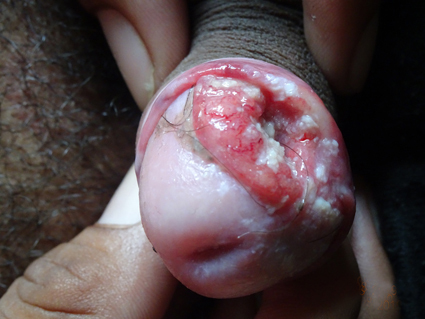
Ulceroproliferative growth measuring 3 × 2 cm over the glans penis. The surface shows purulent discharge and fine vessels.

**Figure 4:**
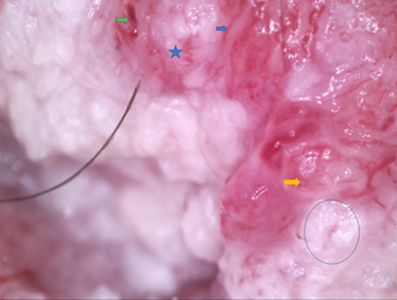
Dermoscopy shows a white structureless area (blue circle), linear vessels (blue arrow), hairpin vessels (yellow arrow), corkscrew vessels (blue star) and blood spots (green arrow) (Dino-Lite Edge, polarized ×100).

A 54-year-old male, who is a chronic bidi smoker, presented with a painless ulceroproliferative growth over the glans penis of 6 months’ duration. The examination revealed an exophytic growth over the glans penis measuring 3 × 2 cm over the superior aspect of the glans and under the surface of the prepuce (Figure 3). Dermoscopy showed white structureless area, white clods, blood spots, erosion, and polymorphic vessels (Figure 4).

**Figure 5:**
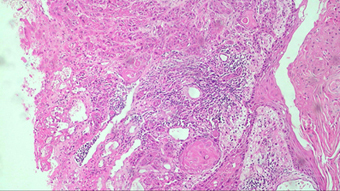
Histopathology shows nests and sheets of tumor cells showing nuclear pleomorphism, dilated vascular channels, and keratin pearl formation (H&E, ×10).

Histopathology in both cases was consistent with moderately differentiated squamous cell carcinoma (SCC) (Figure 5).

## Conclusions

Dermoscopy of invasive SCC shows the presence of keratin and vascular features. Keratin appears as white-to-yellow structureless areas, white circles (opaque center surrounded by a white halo), and white clods. The white circles correlate histopathologically with dilated infundibulum filled with keratin and the white clods to intraepidermal keratin pearls. The amount of keratin depends on the degree of differentiation. Vascular features that can be seen are dotted vessels, glomerular vessels, linear irregular, hairpin, comma, or corkscrew vessels. In the event that one type of vessel morphology predominates, it is either monomorphic or polymorphic. The vessels can have specific arrangements, such as in the clustered arrangement in erythroplasia of Queyrat, which is the genital counterpart of Bowen disease [2]. The vessel morphology depends on tumor volume; dotted vessels are seen in intraepidermal carcinoma, while invasive SCC shows linear vessels. Other features are erosion, blood spots, and crust. The specificity of the white circle for diagnosis of cutaneous SCC is 87%, but it may not be visible on sites where hair follicles are absent in patients with SCC of the glans. Though keratin and vascular features on dermoscopy are common in all forms of SCC, the specific dermoscopy features of white circles are not visible in SCC of the glans, and diagnosis rests on the presence of vascular features and white clods.
